# Detecting Cannabis Use on the Human Skin Surface via an Electronic Nose System

**DOI:** 10.3390/s140713256

**Published:** 2014-07-23

**Authors:** Andreas Voss, Katharina Witt, Tobias Kaschowitz, Wolf Poitz, Andreas Ebert, Patrik Roser, Karl-Jürgen Bär

**Affiliations:** 1 Department of Medical Engineering and Biotechnology, University of Applied Sciences Jena, Jena 07745, Germany; E-Mails: katharina.witt@fh-jena.de (K.W.); Tobias.Kaschowitz@gmx.de (T.K.); doppel.poitz@web.de (W.P.); 2 Department of Psychiatry, Psychotherapy and Preventive Medicine, University of Bochum, LWL University Hospital, Bochum 44801, Germany; E-Mails: Andreas.Ebert@wkp-lwl.org (A.E.); patrik.roser@gmail.com (P.R.); 3 Department of Psychiatry and Psychotherapy, University Hospital Jena, Jena 07743, Germany; E-Mail: karl-juergen.baer@med.uni-jena.de

**Keywords:** electronic nose, principle component analysis (PCA), support vector machine (SVM), pattern recognition, human body odor

## Abstract

The most commonly used drug testing methods are based on the analysis of hair and urine using gas chromatography-mass spectrometry, liquid chromatography-mass spectrometry or immunoassay screening. These methods are time-consuming and partly expensive. One alternative method could be the application of an “electronic nose” (eNose). We have developed an eNose to detect directly on the human skin surface metabolic changes in the human body odor caused by cannabis consumption. Twenty cannabis-smoking and 20 tobacco-smoking volunteers were enrolled in this study. For the sensor signal data processing, two different methods were applied: Principle component analysis (PCA) with discriminant analysis, and the method of pattern recognition with subsequent support vector machines (SVM) processing. The PCA analysis achieved a correct classification of 70%, whereas the SVM obtained an accuracy of 92.5% (sensitivity 95%, specificity 90%) between cannabis-consuming volunteers and tobacco-smoking subjects. This study shows evidence that a low-cost, portable and fast-working eNose system could be useful for health protection, security agencies and for forensic investigations. The ability to analyze human body odor with an eNose opens up a wide field for diagnosing other drugs and also various diseases.

## 1. Introduction

The consumption of substances derived from cannabis such as hashish and marijuana, particularly in the form of joints, is widespread. Cannabis, also called Indian hemp, is a form of the common hemp, a grassy plant with sticky leaves. It contains about 60 chemical compounds with psychoactive effects—the cannabinoids. The main compound is THC (Δ^9^-tetrahydrocannabinol) that is mainly responsible for its psychoactive effects. The relatively high incidence of cannabinoid detection in urine reflects the high prevalence of cannabis use, especially among young adults [1]. Several instrumental analyses techniques for drug testing using different specimens have been reported recently. Urine testing is the most highly developed and most commonly used monitoring technique in substance abuse treatment programs [2]. Alternatively, there has been a growing interest in using blood, hair, sweat or saliva analyses [3,4].

The requirements on such drug-detecting technologies are those of high sensitivity and selectivity for the specific drug to be tested. Furthermore, they should be easy to handle and be able to provide rapid test results. Hair has been used as an alternative specimen to blood or urine for documenting the use of or exposure to drugs [5] since hair analysis can provide information on drug intake for a long time period after the drug has been eliminated from the body. Hair is preferable as a biological sample because of its stability and ease in sampling and storing, compared to conventional biological samples such as blood and urine.

The use of oral fluid (OF) as an alternative matrix for detecting drug abuse has increased over the last decade, leading to the need for a rapid, simple, and reliable on-site OF testing device. Vanstechelman *et al.* [6] evaluated four oral fluid drug-testing devices (Dräger DrugTest 5000, Cozart DDS, Mavand Rapid STAT and Innovacon OrAlert) on 408 volunteers at drug treatment centers. In this study, basic drugs such as cocaine, opiates, tetrahydrocannabinol (THC) as the major species present from cannabis use and some additional ones were considered. In the process of detecting THC, DrugTest 5000 achieved the highest sensitivity (81%), followed by the other devices (23%, 43%, and 28%). In a similar study [7], DrugTest 5000 showed a comparable result.

An electronic nose system (eNose) could be a low-cost, portable and non-invasive alternative for drug testing [8]. An eNose is an instrument which comprises an array of electronic chemical sensors with partial specificity and an appropriate pattern-recognition system capable of recognizing simple or complex odors [9]. The sensing elements are gas sensors with different sensitivities and selectivities. Over the years, several commercially available sensor types have been developed that are specially designed for research purposes. Regardless of the sensor type, physical and chemical interactions take place between the chemical compound and the sensor surface. The most widely used gas sensors are metal oxide gas sensors (MOS), piezoelectric crystal sensors (PCS) and conducting polymer sensors (CPS) [10].

During the past few decades, MOS gas sensors, especially semiconducting MOS (SMOS) sensors, have become a prime technology in several domestic, commercial, and industrial gas-sensing systems [11]. The reacting gas species interacts with the surface of the metal oxide [12]. This relates specifically to the adsorbed oxygen species and the way in which the oxidation of reducing gases (such as CO and hydrocarbons) or the adsorption of oxidizing gases (such as NO_2_ and O_3_) takes place. As a consequence of this surface interaction, a charge transfer takes place between the adsorbed species and the semiconducting sensitive material. The conduction occurring in the sensitive layer is based on the transformation of the sensing process into a measurable electrical signal. This depends strongly on the morphology of the sensitive layer. The change of the concentration of the free charge carriers is transformed into a change in the overall resistance of the sensing layer which indirectly represents the volatile compounds.

Among the available gas-sensing methods, the SMOS gas sensor devices have several unique advantages which include their low cost, small size, measurement simplicity, durability, ease of fabrication and low detection limits (<ppm levels). In addition, most SMOS-based sensors tend to be long-lived and somewhat resistant to poisoning. For these reasons they have rapidly grown in popularity, becoming the most widely used gas sensors currently available [13].

Electronic nose systems have especially been established in the food and pharmaceutical industries and are also used in the military. Recently, eNoses have been developed for medical applications. Volatile organic compounds (VOC) that may be used as non-invasive markers were analyzed from different specimens. *Mycobacterium tuberculosis* could be detected in clinical specimens which are suitable for diagnosing respiratory infections [14]. A novel non-invasive method for diabetes diagnosis based on an electronic nose was proposed by Wang *et al.* [15]. The alteration of body odor in schizophrenic patients was confirmed by GC-MS and chemical sensor array [16]. The results of this study showed that the alteration is complex and cannot be limited to a single compound, and seems to indicate a global variation in human body odor. Other applications proved to detect microbial contaminants in urine samples for the diagnosis of urinary tract infections [17] and identified six species of bacteria responsible for eye infections in saline solutions [18]. Most recently, eNoses have been employed in breath analysis. The detection of lung cancer [19,20], asthma [21] and uremia [22] are especially predominant applications in this regard. Furthermore, human body odor analysis directly from the skin surface was performed to distinguish between different stages of renal dysfunction and based on patients diagnosed with renal diseases and healthy patients as controls [23]. A portable electronic nose system for the identification of cannabis-based drugs was already developed by Haddi *et al.* [8]. However, they investigated the drugs directly as opposed to the metabolites occurring after drug use. The objective of this study was to develop and apply an eNose to distinguish on-site between regular tobacco- and cannabis use by analyzing human body odor directly from the skin surface.

## 2. Methods

### 2.1. Electronic Nose Set up

**Figure 1.**
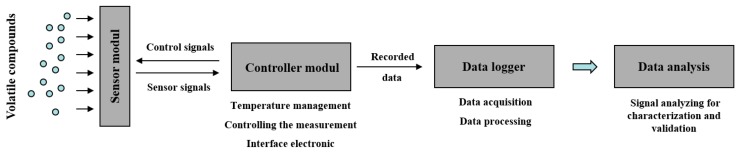
Configuration of the used electronic nose system.

The eNose (Figure 1) consists first of all of an applicator carrying the specific sensor array that is applied to the skin surface. We used customized sensors from the company UST Geschwenda GmbH (Geschwenda, Germany). These multiple SMOS from UST are produced using hybrid technology. The ceramic carrier substrate/chip (Al_2_O_3_) has a dimension of L × W × H: 2.0 × 2.3 × 0.65 mm with platinum thin-film-microstructures for electrodes, heater and contacts. The circuit contains three gas sensitive layers, the GGS1000 series (a specific SnO_2_-compound, thick-film, wideband, especially suitable for combustible gases), GGS3000 series (a specific SnO_2_-compound + Pd-catalyst; thick-film, for hydrocarbons, optimal especially for C_1_…C_8_) and GS7000 series (specific SnO_2_-compound, thin-film for NO_2_). The sensor is the reactive part of the measuring system where each layer of the sensor possesses different sensitivities and selectivities for a variety of different gases at varying temperatures. Therefore, the operating temperature is adjustable between 200 °C and 400 °C. On the sensor surface, a type of interaction is ensured between gas molecules and these sensor layers by changing their conductivity. These changes are measured over time. The sensor material consists of semiconducting oxides (SiO_2_) applied to a ceramic carrier substrate, incorporating a plate heater to control the sensor temperature. By modifying the effective sensor layer and the chip temperature, it is possible to vary the types of gases that can be detected as well as the sensor's sensitivity, although certain physical and chemical limitations can apply. The measuring range, depending on the gas type, ranges from a few ppb to the volume %-range. Although each sensor layer shows a certain degree of affinity towards a specific gas or volatile compound, metal oxide sensors are sensitive towards a wide spectrum of gas species with a somewhat wide overlapping of sensitivities.

The sensor unit is followed by a control unit responsible for controlling the temperature of the sensor heating system and for handling the data recording and data storage. Finally, a data logger collects the sensor data (sensor curves) and specific patient data; this logger contains also a reference data base for specific sensor curves (not used in this study). Data preprocessing and analysis can be performed either on the data logger or on a separate computer.

### 2.2. Data Preprocessing and Analysis

All data preprocessing and data analyses were performed using MATLAB R2009b; statistical analyses were performed either with MATLAB (The MathWorks, Inc., Natick, MA, USA) or SPSS 19 (IBM, Armonk, NY, USA).

**Figure 2.**
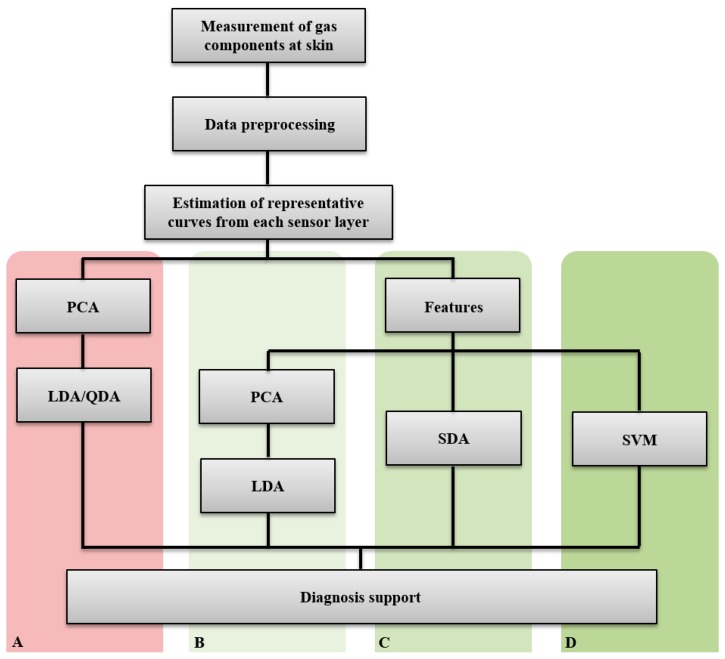
Scheme of data preprocessing and analysis (PCA—Principle Component Analysis; LDA/QDA—Linear/quadratic discriminant function analysis; SDA—Stepwise discriminant function analysis; SVM—Support vector machines; A, B, C, D—data analysis methods).

Sensor data (changes in conductance over different heating temperatures) were interpolated to obtain equidistant temperature values. These values were further averaged to get representative curves for each sensor layer. The data and statistical analyses are divided into two main parts and methods (Figure 2):

1. Analysis of the sensor curves (A)
2. Analysis of features extracted from the sensor curves (B, C, and D)

The first method A directly analyzes the three sensor signals using principal component analysis (PCA), followed by discriminant function analysis (linear -LDA- and/or quadratic -QDA-). The second method is based on extracted features (time- and frequency domains) from the sensor curves and uses as classification methods either B, PCA followed by LDA/QDA, or C, stepwise discriminant function analysis (SDS), or D, support vector machines (SVM).

#### 2.2.1. Feature Extraction from the Sensor Curves

**Figure 3.**
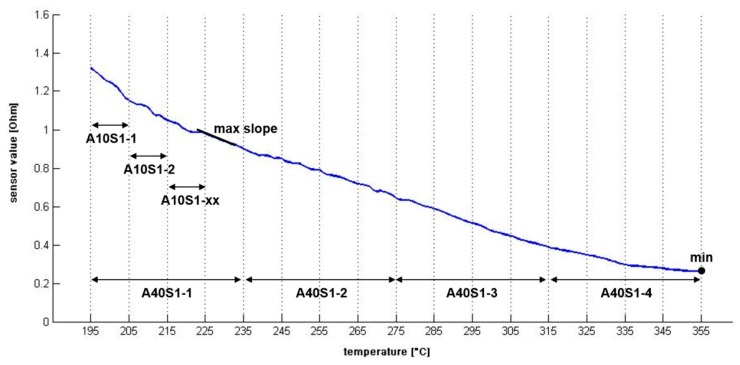
Extracted indices from an averaged (representative) sensor curve.

Several indices (features) from the preprocessed sensor curves were calculated (see Table 1). Such indices from the time domain are total areas and subareas (to view examples, see Figure 3). Further on, the steepest slope and the corresponding temperature within the curve were calculated, respectively.

**Figure 4.**
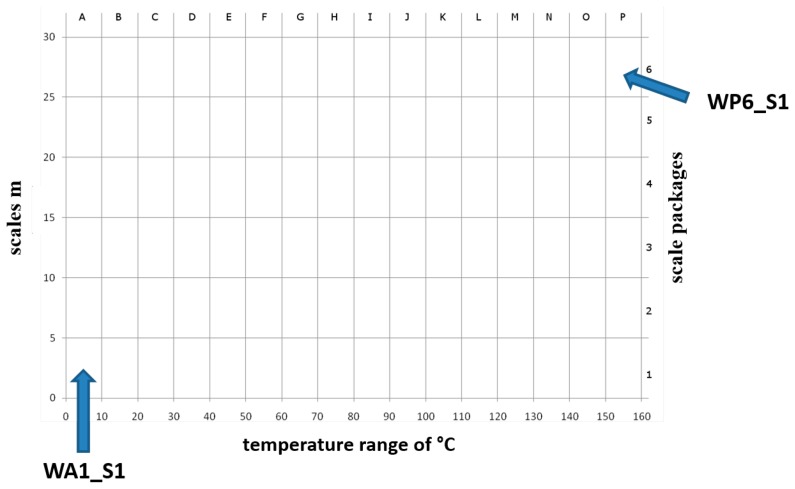
Scheme of WT scaling diagram (the temperature range in absolute values is 195–355 °C). As examples WA1_S1 means the summarized power in the scales 0–5 (scale package 1) over a temperature range of 0–10 °C (relative degrees, absolute degrees: 195–205 °C) and WP6_S1 stands for the summarized power in the scales 25–30 (scale package 6) over a temperature range of 150–160 °C (relative degrees, absolute degrees: 345–355 °C).

The continuous wavelet transform (WT) decomposes a signal into time-frequency space [24]. It was applied to estimate frequency domain indices whereby the Daubechies wavelet was used. The scales *m* were summarized at a 5-scale interval and at a 10 °C temperature interval (e.g., WA1_S1: summarized scales 0–5 over a temperature range of 0–10 °C (relative degrees, absolute degrees: 195–205 °C). Figure 4 shows the resulting scaling diagram. All extracted indices are listed in Table 1.

#### 2.2.2. Principal Component Analysis

Principal component analysis (PCA) is a multivariate projection- and dimension reduction technique. This method provides the meaning of a type of data set by extracting a smaller series of important components that account for the data variability. Each of these factors or principal components considers a subset of the variables to be important. The principal components are obtained through a linear combination of the dependent variables that maximizes the variance within the data set. The first principal component accounts for the largest amount of data variation. The second principal component is not correlated to the first and is orthogonal to the first principal component and accounts for the second largest remaining variation in the data. The other principal components are generated using the same criteria [25,26].

The results from PCA were reduced to their first and second principal odor components. These components were plotted in two-dimensional graphs to visualize between-group separations. Once these components were calculated, they were used to perform a linear LDA or QDA, the LDA (as simpler version) being preferred.

#### 2.2.3. Discriminant Analysis

Discriminant analysis (DA) allows for distinguishing between groups based on certain predictor variables. The mathematical function that combines information from predictor variables in order to obtain the maximum discrimination among groups is called “discriminant function” [27]. QDA performs the calculation of the discriminant score not by one covariance matrix, but by covariance matrices separately estimated for each class. The discriminant function is a quadratic function and contains second order terms.

SDA is an attempt to find the best set of predictors. In SDA, the most correlated independent index (variable) is entered first by using the stepwise procedure followed by the next one until an additional dependent adds no significant amount to the discrimination power. The criterion for adding or removing is typically based on the setting of a critical significance level. SDA is applied directly onto the extracted features to obtain the maximum separation between the groups (Test C).

#### 2.2.4. Support Vector Machines

The support vector machine (SVM) is a novel type of learning machine based on the statistical learning theory for two-group classification problems [28,29]. Input vectors are non-linearly mapped to a very high dimension feature space within which a linear decision surface is constructed. The conceptual part of this problem was solved for the case of *optimal hyperplanes* for separable classes.

The advantages of margin maximization are best achieved by mapping these points into a high-dimensional feature space where a separating hyperplane is located which maximizes the margin. Hyperplanes can be represented in feature spaces by means of kernel functions. In this study, the radial basis kernel (radial basis function RBF) is used.

The optimal hyperplane is then constructed within the feature space creating nonlinear boundaries in the input space. The decision function is formulated in terms of these kernels. The choice of the appropriate penalty parameters C and the kernel characteristic parameter σ is essential in obtaining a well-tuned SVM [28,29].

During the first step of the SVM application, optimum C and σ with different combinations (C 1–1000, σ 0.1–50) were estimated separately for the time domain- and frequency domain indices. In the second step these optimized C and σ are used to obtain the best separating 10 indices, including cross-validation. The final result consists of cumulative sensitivities and specificities for these 10 indices.

### 2.3. Patients and Study Design

The study population was recruited from the LWL-University Hospital of Bochum and included two groups of healthy volunteer subjects. The one group consisted of 20 subjects who smoke tobacco; the other group consisted of 20 subjects who use cannabis. Smokers were defined as subjects who had smoked regularly on some or all days in the previous month and cannabis smokers as subjects who had used cannabis regularly on some days per week or at least two times a week in the previous month. Table 2 shows the characteristics of the study population.

All subjects did not smoke tobacco or cannabis at least 12 h before investigation. The data were recorded in the same room under comparable conditions during the morning hours. The sensor head was placed in the crook of the arm. Before starting a complete measurement, the skin surface was prepared, with the reason of avoiding any contaminating gas component from the subjects' application of personal hygiene products. Sensor curves were sampled 10 times over 30 min (one measurement period lasted 3 min). The sensor layers were recovered (heating the layers to 360 °C for 5 min) before each patient measurement. Before the measurement procedures began, each subject underwent a urine test that checked for drug consumption. This study met the recommendations of the Declaration of Helsinki. Furthermore, the ethical committee of Bochum University approved the study protocol, and all study participants gave their written informed consent before study participation.

## 3. Results and Discussion

### 3.1. Applying Linear and Quadratic LDA/QDA on PCA Results (A)

As aforementioned, PCA was performed on the representative sensor curves. The results from the PCA analysis were reduced to the first and second principal odor components containing the maximum variance found in the data. Then, LDA was applied on these PCA results to separate between the main principal odor components found in both groups. The PCA analysis, in combination with LDA analysis of the three sensor signals, achieved an accuracy of 67.5% (see Table 3) discrimination between cannabis-using subjects and tobacco smokers.

### 3.2. Applying Discriminant Analysis on PCA Results from Sensor Curves' Extracted Features (B)

LDA was applied on PCA results from extracted features of the different sensor curves. The LDA achieved a maximum sensitivity of 80%, a specificity of 50% and a total accuracy of 65% (see Table 3).

### 3.3. Applying Discriminant Analysis on Extracted Features of Sensor Curves (C)

The SDA was applied directly to the extracted features of sensor curves to obtain the maximum separation level between the two groups. The SDA achieved a sensitivity of 80%, a specificity of 85% and a total accuracy of 82.5% (Table 3) by means of four indices from the time- and frequency domain (WC6_S3, WE5_S1, WK6_S3, and Tmax_slope_3_S1).

### 3.4. Applying SVM on Extracted Features of Sensor Curves (D)

The SVM analysis was divided into two steps. First, a total of 87 indices from only the time domain and, second, a total of 288 indices from the frequency domain, were used as SVM input data. For each type of classification, the optimal parameter set including up to a maximum of 10 indices were considered.

**Figure 5.**
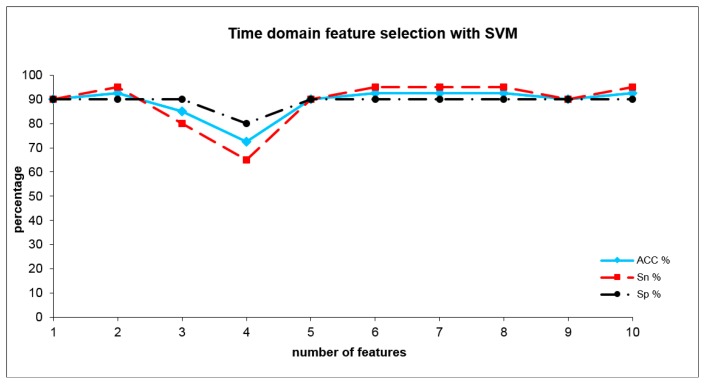
Accuracy, sensitivity and specificity using SVM analysis from time domain indices where the group cannabis use- was discriminated from the group tobacco smokers group, dependent upon the number of included optimum indices.

The optimum adjustment parameters of the SVM for time domain analysis were set to C = 10 and σ = 20, with an error margin of 0.4. The SVM (see Table 3) reached a sensitivity of 95% and a specificity of 90% and a total accuracy of 92.5%, already with only two time domain indices representing subareas. These indices were: A10S2_10, A10S3_1. The development of SVM results where the number of included indices was increased is shown in Figure 5.

**Figure 6.**
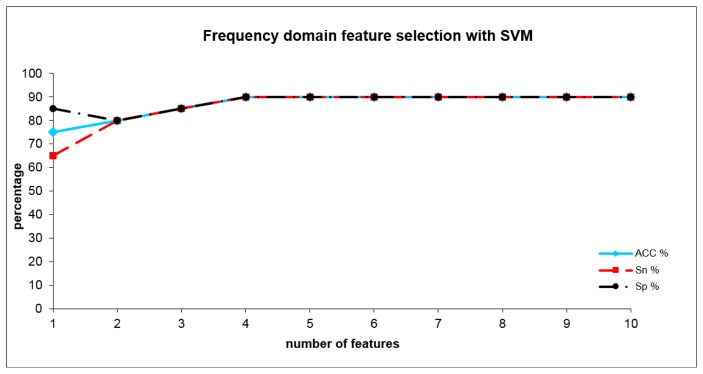
Accuracy, sensitivity and specificity using SVM analysis from frequency domain indices where the cannabis-users and tobacco-smokers group could be discriminated depending on the number of included optimum indices.

The optimal SVM adjustment parameters for the frequency domain analysis were set to C = 1 and σ = 0.05 with an error margin of 0.4. The SVM (see Table 3) reached a sensitivity of 90%, a specificity of 90% and a total accuracy of 90% with four frequency domain indices. These indices were WH6_S1, WN4_S1, WJ2_S1 and WO2_S2. Figure 6 shows the development of SVM results when the number of included indices are increased. The combination of time- and frequency domain features did not outperform the results from the time domain alone.

In a previous study [30] we already investigated the general influence of tobacco smoking on data recorded with this eNose. Here the sensor was placed (in the same manner as described in the Methods section) on skin and in parallel the exhaled breath was analyzed. The differentiation between 11 smokers and 11 non-smokers (age and gender matched) revealed a correct separation for skin application 96% (only one misclassified case) and (not surprisingly) for exhaled breath application 100%. As biomarker we could clearly detect furan using GC/MS that was also the strongest discriminator in the sensor curves of the eNose in both applications.

In summary, the aim of this pilot research study was to apply a mobile electronic nose to screen for the use of cannabis in humans by analyzing the odor compounds found directly on human skin. Using two indices from the time domain, we were able to discriminate a group of cannabis users from a group of normal tobacco smokers with an accuracy of 92.5%.

Cannabis is the illicit drug with the highest worldwide consumption prevalence and the highest rate of positive findings in workplace drug testing [31]. Cannabis is a drug that might lead to addiction and significant negative consequences. A speedy recognition of cannabis use is therefore of great interest for various applications, including post mortem tests.

The applied electronic nose system allows one to indirectly detect and classify odor compounds, and therefore could be used as a tool for diagnosing different diseases [23]. By analyzing sensor signals, one detects similarities and differences between the odor compounds of different subjects, different groups or different time instants. The PCA analysis is the standard classification method with a high potential to differentiate between two stages. In this study, however, we could show that the SVM has some advantages over PCA when discriminating cannabis users from tobacco smokers. By generating a hyperplane on the basis of the statistical learning theory, it is possible to maximize the level of discrimination accuracy between different classes. This method also presents the possibility to analyze complex data such as ours. The successful application of SVM in conjunction/association with an electronic nose system was already confirmed in several studies in other fields [32].

Human body odor is generated by waste materials present on the skin surface and secretions from the sweat- and sebaceous glands. These waste materials are converted to characteristic odorous compounds through oxidative degradation or through the metabolism process of skin microbes. Changes in body odor due to aging relate to the amount and composition of sweat and sebum secreted as well as to gland activity [33]. Metabolic changes in the body are associated with typical odors which can be measured in the breath [34], sweat [35] or other human excreta [16], and can be used as biomarker(s) for a specific disease [36]. Several reports have demonstrated that sweat is a suitable alternative biological matrix for monitoring recent drug use. This is based on the assumption that, in the context of the absorption, distribution, metabolism or excretion cycle of drugs, a small but sufficient fraction of a drug is excreted into the sweat and can be tested [31].

Even though metal oxide semiconductor sensors are sensitive towards a wide spectrum of gas compounds, we must consider that the sensor signals provide only information about changes in conductivity in relation to the sensor temperature. One limitation is that they do not provide any information about the concrete characteristics of the existing odor components. Therefore, a reference method of analysis should be used in order to find the concrete biomarkers. The GC/MS analysis is a method that enables chemical compounds to be identified [37]. An identification of the chemical components (biomarkers) within the gas samples would offer an enhancement to the sensor's characteristics (specific dotation of the sensor layers) and would lead to a further increase in sensitivity and specificity of the eNose. In a previous study, we could prove that the GC/MS analysis is an efficient method for the chemical characterization of breath compounds [30].

Limitations of this pilot study are: (a) we did not yet estimate the concrete biomarkers responsible for discriminating between tobacco and cannabis on skin. Therefore, in an ongoing study, the GC/MS analysis method will be performed to characterize the cannabis drug's specific biomarkers on the skin surface; (b) we have not yet clarified the influence of any medications used, individuals' grooming habits, nutrition and environmental influences; (c) furthermore, the correlations between the odor components and clinical parameters [23] need to be analyzed; (d) Finally, it should be noted that in this study only a relatively small number of subjects were enrolled in the two groups. However, this was only a pilot study to develop the proof of concept and, of course, has to be followed by an enlarged validation study.

In this study we could demonstrate that an electronic nose could discriminate cannabis users from tobacco smokers via human body odors on the skin surface. For this first study, a broadband three-layer metal oxide semiconductor sensor was applied. By applying SVM on features extracted from the sensor curves, we achieved a remarkable accuracy of 92% when discriminating between cannabis and tobacco smokers. The most discriminant features were derived from the time domain, representing areas of conductivity (summarized in 10 °C intervals) related to specific sensor heater temperatures. With the presence of only two of them (A10S2_10, A10S3_1), this discriminating power could be achieved.

Melamede [38] recently published that “smoke from tobacco and cannabis contains many of the same carcinogens and tumor promoters. However, cannabis and tobacco have additional pharmacological activities, both receptor-dependent and independent, that result in different biological endpoints. Polycyclic aromatic hydrocarbons found in smoke are pro-carcinogens that are converted to carcinogens” and “…the THC present in cannabis smoke should exert a protective effect against pro-carcinogens…”. Interestingly, from sensor layer 2 which is sensitive for hydrocarbons one of the two best discriminating features was revealed.

When adding further indices after a short decrease (including 3 and also 4 indices), this accuracy remained practically stable including five or more indices (see Figure 5). By combining four frequency domain indices (WH6_S1, WN4_S1, WJ2_S1 and WO2_S2), we achieved an accuracy of 90%. These indices represent differences within lower and higher scales (in nearly all frequencies) at higher temperatures (shown by the rear part of the sensor curves). When adding further indices, this accuracy level remained stable (see Figure 6). Surprisingly, the combination of time- and frequency domain results did not lead to an improved result (and therefore detailed results are not presented). Even if in the frequency domain the information derived from layers 2 and 3 dominate the discrimination between the groups in the time domain, these are the information from layer1. This result is a sign of the more complex nature of the differentiating odors and suggests a mixture of different gas compounds representing different cannabis metabolites.

Comparing our results with those from other approaches, we found e.g., in a study of Rörich *et al.* [39] that they compared GC–MS analysis of oral fluid with Rapid Stat results (the Rapid Stat assay is a point-of-collection drug-testing device for detecting amphetamines, cannabinoids, cocaine, opiates, methadone and benzodiazepines in oral fluid) with regard to cannabis use. They could show a sensitivity of 85%, a specificity of 87%, and a total confirmation rate of 87%. The development of an analytical method to determine the existence of Δ^9^-tetrahydrocannabinol (THC), cannabidiol (CBD) and cannabinol (CBN) in human hair samples was described by Emídio *et al.* [40]. Using headspace mode solid-phase microextraction in combination with GC/MS, they found in 10 samples CBD and CBN with a detection rate of 100%, and THC (the main psychoactive cannabinoid) with a detection rate of 70%. Similar results (also from hair analysis) were obtained by Backofen *et al.* [41] using the analytical methods of non-aqueous capillary electrophoresis with electrochemical detection.

Considering all of the known established methods of cannabis detection, one can summarize that up until now valid detection for cannabis has been time-consuming. Furthermore, tests cannot determine the approximate degree of health impairment. The lack of suitable tests could be overcome by the application of eNose systems, as introduced in this study. Haddi *et al.* [8] already showed that with a simple, low-cost, portable electronic nose system based on commercially available metal oxide gas sensors, a classification of different types of drugs is possible. However, their study characterized the drugs but did not prove the aspect of their usage by humans. We believe that our method is the first to show that an eNose system could be able to detect cannabis use with a much higher degree of accuracy.

## 4. Conclusions

This study shows that an electronic nose system has the ability to differentiate between human body odor of subjects using cannabis and normal tobacco-smoking subjects. We demonstrate that not only metabolic changes caused by diseases are detectable from the skin surface, but that it is also possible to identify drug (here cannabis) consumption. This study provides evidence that after a necessary validation a low-cost, portable and fast-working eNose system could be useful for health protection and for security agencies.

## Figures and Tables

**Table 1.** Extracted indices from time- and frequency domains (S1—sensor layer1, S2—sensor layer2, S3—sensor layer3).

Methods	Index	Description
**Time Domain**	AS1; AS2; AS3;	total area of each sensor layer curve

	A10S1_1…A10S1_xx;	sub area of each sensor layer with an incremental of 10 °C within curves
	A10S2_1…A10S2_xx;	
	A10S3_1…A10S3_xx;	

	A40S1_1…A40S1_xx;	sub area of each sensor layer with an incremental of 40 °C within curves
	A40S2_1…A40S2_xx;	
	A40S3_1…A40S3_xx;	

	Max_slope_3_Sx and Tmax_ slope_3_ Sx; Max_slope_5 and Tmax_slope_5_Sx	steepest slope of 3/5 points within curves and the corresponding temperature Tmax (Sx: Layer 1..3)

**Frequency Domain**	WA1_S1; WA2_S1… WP6_S1	frequency components of the curves from S1

	WA1_S2; WA2_S2… WP6_S2	frequency components of the curves from S2

	WA1_S3; WA2_S3 … WP6_S3	frequency components of the curves from S3

**Table 2.** Characteristics of the study population.

Groups	Cannabis Smokers	Tobacco Smokers
Age [year]	25.7 ± 3.6	26.6 ± 4.7
Amount of tobacco consumption [cigarettes per day]	11.0 ± 9.8	12.0 ± 6.3
Amount of cannabis use [joints/week]	16.2 ± 12.0	-
Frequency of cannabis use [times per week]	4.8 ± 1.7	-
Duration of cannabis use [years]	7.5 ± 4.1	-

**Table 3.** Summarized results of the discriminated groups cannabis users and tobacco smokers (Test A is performed by first using two principal odor components made apparent from sensor curves and traditional linear discriminant analysis, Tests B, C and D are performed using extracted indices from the sensor curves; test B uses PCA and LDA, test C uses SDA and test D uses SVM for classification and discrimination purposes).

Test	Sensitivity	Specificity	Accuracy
A	85%	50%	67.5%
B	80%	50%	65.0%
C	80%	85%	82.5%
D	95%	90%	92.5%
